# Plan Quality Comparison at Five Years in Two Cohorts of Breast Cancer Patients Treated with Helical Tomotherapy

**DOI:** 10.3390/jcm14051630

**Published:** 2025-02-27

**Authors:** Samantha Dicuonzo, Maria Alessia Zerella, Mattia Zaffaroni, Maria Giulia Vincini, Karl Amin, Giuseppe Ronci, Micol D’arcangelo, Damaris Patricia Rojas, Anna Morra, Marianna Alessandra Gerardi, Cristiana Fodor, Raffaella Cambria, Rosa Luraschi, Federica Cattani, Paolo Veronesi, Francesca De Lorenzi, Mario Rietjens, Roberto Orecchia, Maria Cristina Leonardi, Barbara Alicja Jereczek-Fossa

**Affiliations:** 1 Division of Radiation Oncology, European Institute of Oncology IRCCS, 20141 Milan, Italymariaalessia.zerella@ieo.it (M.A.Z.); mariagiulia.vincini@ieo.it (M.G.V.); marianna.gerardi@ieo.it (M.A.G.);; 2 Department of Oncology and Hemato-Oncology, University of Milan, 20141 Milan, Italy; 3 Unit of Medical Physics, IEO, European Institute of Oncology IRCCS, 20141 Milan, Italyrosa.luraschi@ieo.it (R.L.); federica.cattani@ieo.it (F.C.); 4 Division of Breast Surgery, European Institute of Oncology IRCCS, 20141 Milan, Italy; 5 Division of Plastic and Reconstructive Surgery, IEO, IRCCS European Institute of Oncology IRCCS, 20141 Milan, Italy; 6 Scientifc Directorate, European Institute of Oncology IRCCS, 20141 Milan, Italy

**Keywords:** intensity-modulated radiotherapy (IMRT), plan quality, breast cancer, post-mastectomy radiotherapy (PMRT)

## Abstract

**Objectives:** this study aimed to evaluate the evolution of planned dose distribution quality in two groups of breast cancer patients treated with hypofractionated intensity-modulated radiotherapy (IMRT) using Helical TomoTherapy^®^ at our institute 5 years apart. **Methods**: the analysis included two cohorts of patients who underwent implant-based immediate breast reconstruction (IBR) and received post-mastectomy IMRT to the chest wall and infra/supraclavicular lymph nodes, following a 15-fraction regimen (2.67 Gy per fraction). The first group was treated between 2012 and 2015, while the second received treatment between 2019 and 2020. Dosimetric indices derived from dose–volume histograms used in clinical practice were analyzed to assess dose distribution quality. A quantitative scoring system was applied retrospectively to compare the two groups in terms of target coverage and organ-at-risk (OAR) sparing. Additionally, capsular contracture (CC) incidence was examined in both cohorts. **Results:** A total of 240 patients were included in the study. The percentage of optimal treatment plans increased from 70.8% in the 2012–2015 cohort to 77.5% in the 2019–2020 cohort, while compromised plans decreased from 10.8% to 7.5%. Furthermore, the incidence of moderate-to-severe CC dropped from 54.8% in the earlier cohort to 43.5% in the later one. **Conclusions:** Helical Tomotherapy^®^ has demonstrated the ability to achieve a high rate of optimal treatment plans concerning both PTV coverage and OAR sparing in a challenging population of postmastectomy patients with IBR. The learning curve showed that, after 5 years, the rate of optimal plans was increased, accompanied by a reduction in compromised plans and treatment-related toxicity.

## 1. Introduction

Postmastectomy radiotherapy (PMRT) has been proven effective for local control and survival rates in patients with advanced and intermediate-risk breast cancer (BC) [1].

However, many patients undergo immediate breast reconstruction (IBR) after radical surgery, with either a permanent implant (PI) or a temporary tissue expander (TE). These reconstruction methods can interfere with PMRT to varying degrees [2], resulting in technical challenges in treatment planning.

Advanced techniques, like intensity-modulated radiotherapy (IMRT), have been shown to address these issues, allowing for precise adaptation of radiation fields to the geometry of the reconstructed breast and providing a better dose homogeneity and lower complication rates [3,4].

In the setting of PMRT, with or without IBR, chest wall (CW) and regional lymph node irradiation with daily fractions of 1.8 to 2.0 Gy are usually delivered, for a total dose of 45 to 50.4 Gy in 25 to 28 fractions.

Currently, moderate hypofractionation (15 fractions) is still not widely used for PMRT, especially in the presence of implants, because of concerns about safety for adjacent critical organs (OARs) and cosmetic outcomes; however, there is a growing interest in its use, and recent prospective and retrospective studies have shown similar recurrence rates and no significant difference in acute or late toxicities between conventional and hypofractionated radiation therapy [5,6,7,8].

Moreover, in the recently published consensus by the European Society for Radiotherapy and Oncology (ESTRO), moderate hypofractionated PMRT has been established as the recommended scheme, also in the case of IBR [9].

Usually, when a standard treatment changes, more expertise is required. For instance, previous studies about whole-brain irradiation have shown that the crucial element in hippocampal-sparing brain radiotherapy treatment planning is the appropriate contouring of the hippocampus, underlining the importance of the so-called process of “learning curve” [10]. Similarly, in the surgical setting, a number of studies have systematically analyzed the learning curve of new approaches: in the minimally invasive lobectomy, several parameters concerning the quality, the effectiveness, and the radicality of the surgical act have been considered and there are already many studies on the learning curve for minimally invasive liver surgery [11,12].

On the other hand, to the best of our knowledge, in the setting of PMRT treatment planning, no standardized parameters are available to systematically describe and evaluate the learning process of implementing a new radiotherapy (RT) scheme (i.e., moderate hypofractionation). In such a scenario, a quantitative scoring tool, comparing differences between RT treatment plans alongside two time points, could be a reasonable method to analyze the expected implementation of RT planning by physicists [7].

The aim of this study was to evaluate the dosimetric quality of radiation treatment plans for hypofractionated PMRT with IMRT delivered using Helical Tomotherapy^®^ (Accuray Incorporated, Sunnyvale, CA, USA) after implant-based IBR in a monocentric and retrospective series. In particular, the learning curve in this setting will be assessed by comparing the quality of delivered RT plans between 2012–2015 and 2019–2020.

## 2. Materials and Methods

A total of 240 breast cancer patients (half treated between 2012 and 2015 and half treated between 2019 and 2020) with implant-based IBR who received PMRT to the chest wall (CW) and the infra/supraclavicular nodal region (SVC) using a 15-fraction schedule (2.67 Gy/fraction) at the European Institute of Oncology (IEO, IRCCS, Milan, Italy) were included in the study.

Patients received mastectomy, sentinel lymph node biopsy with or without axillary dissection, and IBR with either a TE or PI for stage II and stage III breast cancer. The TE/PI was placed behind the pectoralis major muscle and the serratus anterior muscle or its fascia based on the mastectomy flap thickness and blood supply, the location of the surgical scar, and the extent of skin removal. The institutional ethical committee approved all the analyses and all patients provided written informed consent for the treatment and anonymous use of their data for educational and research purposes.

### 2.1. Radiotherapy Treatment Technique

Axial computed tomography (CT) images with a slice thickness of 2.5 mm, including neck, chest, and upper abdomen, were acquired in supine patients lying on a breast board and used for treatment planning.

**Figure 1:**
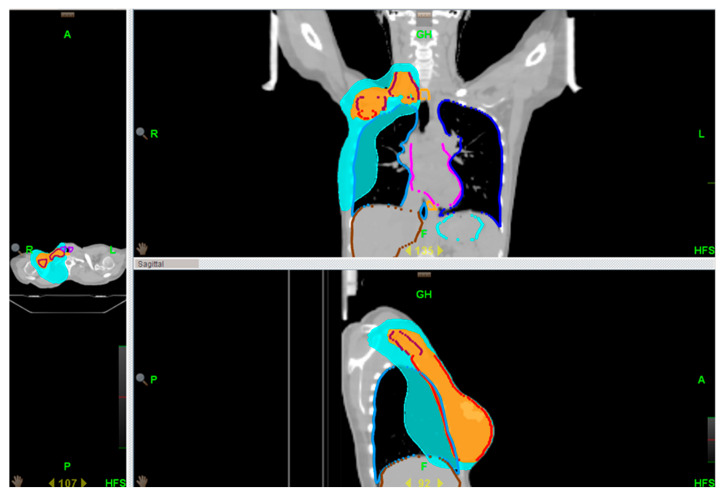
An example of a locoregional treatment plan.

Target volumes and OARs were contoured according to both national guidelines and the breast cancer atlas of the Italian Association of Radiotherapy ad Clinical Oncology (AIRO) and ESTRO [13,14]. Contouring and treatment planning was performed using Eclipse^®^ version 8.6 (Varian Medical Systems, Palo Alto, CA, USA) and Raystation version 11a (RaySearch Laboratories AB, Stockholm, Sweden). Planning was performed for both cohorts in TomoTherapy Volo^TM^. The clinical target volumes (CTVs) comprised the chest wall, level II–III axillary nodes, and the supraclavicular region (Figure 1). In accordance with standard clinical practice, the temporary TE was fully inflated prior RT. When clinically needed (e.g., for skin involvement or inflammatory tumors), a 5 mm tissue equivalent bolus was applied for half a treatment to increase the surface dose.

The prescribed radiation dose was 40.05 Gy in 15 fractions (2.67 Gy per fraction) over a 3 week period. The planning target volume (PTV) included the CW-CTV with an isotropic margin of 5 mm and the regional node (SVC)-CTV with anisotropic margins (0 mm contralaterally and posteriorly and 5 mm craniocaudally, anteriorly, and medially), to minimize the exposure of esophagus and brachial plexus. PMRT was delivered using the helical modality of the TomoTherapy^®^ Hi-Art System (Tomotherapy^®^ System, Accuray Incorporated, Sunnyvale, CA, USA) in helical mode. A jaw width of 2.5 cm was used with a pitch of 0.430 and modulation factors of 1.8–2.0, ensuring a delivery time of approximately 10–15 min. Throughout the optimization, dose–volume histogram (DVH) points and penalties were adjusted to best meet OAR dose constraints while maintaining adequate PTV coverage. Daily setup verification was image-guided.

### 2.2. Treatment Plan Evaluation and Scoring

Treatment plans were evaluated according to common dosimetric DVH-derived indices. A quantitative scoring tool, adapted from the one used by Motwani et al. [15], was used to retrospectively assess the quality of the planned dose distribution between the two sub-cohorts, taking into account both coverage of the target and the unwanted dose to the OARs. Details about the scoring system were previously published [7]. Comprehensively, the plan was deemed optimal if it scored a total of 6 points and acceptable if it received 5.5 points. Plans awarded less than 5.5 points were considered compromised.

Dosimetric indices between the two cohorts (2012–2015 vs. 2019–2020) were compared using non-parametric two-tailed Mann–Whitney U test and the significance level was set to *p* < 0.05.

### 2.3. Toxicity Evaluation

For toxicity evaluation, data about capsular contracture (CC) were censored at the last clinical evaluation by a radiation oncologist/plastic surgeon or implant removal. Degrees of CC were graded by the Baker classification [16]. Reasons and rates for eventual implant removal were collected and reported.

## 3. Results

### 3.1. Plans Evaluation

The distribution of total scores are reported in Table 1.

An improvement in the plan quality was observed along the two time points with the percentage of optimal plans increasing from 70.8% in 2012–2015 to 77.5% in 2019–2020 (+6.7%), while the percentage of compromised plans decreased from 10.8% to 7.5% (−3.3%).

Median values of dosimetric indices, as well as the percentage of plans satisfying each planning objectives, both for the individual organs and target volumes, are reported in Table 2.

The percentage of appropriated plans increased for the most part of considered constraints.

Moreover, considering CW-PTV and SVC-PTV, a significant increase of V95% and V90% in 2019–2020 plans was observed.

Regarding OARs, the dose received by the ipsilateral lung was significantly lower in the 2019–2020 plans, while a slight increase (*p* < 0.05) in dosimetric indices along the two time points was found for the brachial plexus, spinal cord, and esophagus, but still well below dose constraints.

### 3.2. Toxicity Evaluation

In the cohort receiving PMRT between 2012 and 2015, at least one evaluation with the plastic surgeon and/or radiation oncologist was available for 109/120 patients, with a median follow-up of 24 months (range 0–90 months); data about capsular contracture was recorded for 84/109 patients. The assessment of CC was lost for 24 patients and not assessable for 1 patient.

Moderate (Baker III) and severe (Baker IV) CC was recorded in 40.5% and 14.3% of the patients, respectively.

Likewise in the cohort treated more recently (2019–2020), plastic surgeon and/or radiation oncologist evaluation was available for 66 patients, of which CC grading following Baker classification was collected for 62 patients; for the remaining 4 patients, Baker assessment was not available. With a median follow-up of 22 months (range 2–41 months), 38.7% and 4.8% of the patients had a Baker III and Baker IV CC, respectively.

Details about the distribution of patients according to Baker classification are reported in Table 3 and details about patients’ characteristics for both cohorts are reported in Table 4.

Interestingly, 31 (28%) of the 109 patients treated between 2012 and 2015 needed an implant substitution (13 for CC, 11 for asymmetry/surgical complications, and 7 for other reasons), while in the 2019–2020 group, only 8 (8%) out of 89 patients underwent an implant substitution (4 for CC, 3 for asymmetry/surgical complications, and 1 for other reasons).

## 4. Discussion

In the current study, we present the results of our retrospective analysis evaluating treatment plans of two groups of BC patients treated at two different time points with hypofractionated IMRT with Helical TomoTherapy^®^ at the European Institute of Oncology (IEO, IRCCS, Milan, Italy).

To the best of our knowledge, this is the first study to assess the learning curve of dosimetric quality of RT plans for hypofractionated PMRT after IBR using Helical Tomotherapy^®^.

The 5-year learning curve has shown a significant improvement in target coverage; regarding OARs, a significant sparing was recorded only for the ipsilateral lung, but all of the OARs dose constraints were respected. At the same time, with a similar follow-up, we observed a reduction in severe toxicity rate in terms of CC. Because the exact cause of CC is still unclear and various risk factors could play a key role in its development, further analyses are needed to confirm a possible correlation between dosimetric parameters as well as clinical characteristics in the present population.

Moderate hypofractionation in the setting of PMRT with IBR is not a standard approach worldwide [17,18,19,20], mainly because of the concerns about reconstruction complications. Nowadays, only one randomized phase 3 trial comparing normo- and moderate fractionation is ongoing, with final toxicity results expected in 2035 [21]. On the other hand, in this setting, retrospective and prospective studies have not shown higher toxicity rates and the recent ESTRO-ACROP (Advisory Committee for Radiation Oncology Practice) consensus recommends this scheme regardless of the presence and the type of reconstruction [9,22,23,24].

At our department, moderate hypofractionation was successfully adopted since 2012 for patients with both prosthesis and temporary TEs, with only 15% of women experiencing a major reconstruction complication [8].

In clinical practice, changing a standard approach (i.e., moving from normofractionation to moderate hypofractionation) could be challenging, but, as we reported in the present experience, the increasing expertise of our medical physicists led to more feasible and better planning over the years, resulting also in a decrease in toxicity considering both CC rates and number of implant substitutions. Consequently, the present study could encourage the introduction of moderate hypofractionation in daily practice representing a useful platform for other clinicians to both compare dose constraints and refine them to optimize helical PMRT.

This study is not exempt from limitations, mainly attributable to its retrospective nature. First of all, an update of follow-up is under evaluation in order to investigate and correlate dosimetric data with further toxicities (i.e., both skin and soft tissue complications, together with CC). The follow-up for CC among the two cohorts was similar (22 vs. 24 months) to avoid confounding related to large differences between the two assessments timepoints. Nevertheless, a difference in the number of patients with a plastic surgeon/radiation oncologist clinical evaluation between the two cohorts was observed (109/120 patients in the cohort from 2012–2015 and 66/120 in the cohort from 2019–2020). Of note, follow-up of the latter group was negatively affected by the COVID-19 pandemic that increased the use of telemedicine with no possibility to collect data about CC. Importantly, the majority of patients continued their evaluations with clinical oncologists/breast surgeons. This notwithstanding, in our daily clinical practice, if an adverse event such as a CC occurs, patients are reasonably motivated to return and strictly attend the follow-up care program with all the specialties of the multidisciplinary team. As a consequence, we can assume that if patients missed appointments, 3–4 Baker CC did not occur with a theoretical improvement of the general results of our study. The present study, being a dosimetric study, lacks correlation of dosimetric data with clinical outcomes; a more clinical study may be of future interest whit more mature follow-up data.

Finally, differences among treatment plans could be related to a variability in the employed/commissioned physicist: in the present experience, five different physicists with more than 5 years of experience in breast cancer treatment planning were involved, but this team did not change between the two analyzed time periods.

A comparison between our series and other similar experiences or results is difficult given the paucity of available literature about treatment planning learning curve in Helical Tomotherapy^®^ and the correlation with toxicity.

However, a step toward a better RT tolerance through an improvement of pre-treatment processes is of great interest: the contouring guidelines endorsed by ESTRO-ACROP breast cancer experts [25] are an example of this kind of approach.

In conclusion, we believe that an improvement in treatment plans could represent a useful tool to test the physician learning curve and that advances in treatment planning could contribute to reducing RT-related side effects, creating the conditions for a change in clinical practice, but further studies are required to validate our hypothesis.

## Figures and Tables

**Table 1:** Total scores determined by summing points for PTV coverage and OAR sparing: 6 points were assigned for optimal plans, 5.5 points for acceptable plans, ≤5 points for compromised plans.

	Total Score (Points)	n Plans 2012–2015 (%) N = 120	n Plans 2019–2020 (%) N = 120	Δ
Optimal	6	85 (70.8)	93 (77.5)	+6.7%
Acceptable	5.5	22 (18.4)	18 (15.0)	−4.0%
Compromised	≤5	13 (10.8)	9 (7.5)	−3.3%

**Table 2:** Median values of dosimetric indices and plans satisfying each planning objectives/constraints. Significant *p* values are shown in bold.

	Median Value (IQR) 2012–2015	Median Value (IQR) 2019–2020	*p* Value	Satisfying Plans 2012–2015 (%)	Satisfying Plans 2019–2020 (%)	Δ
**CW-PTV**						
V95% ≥ 90%	94.9 (92–97)	96.3 (92.6–98.0)	**0.03318**	85.8	90	+4.2
V90% ≥ 95%	99 (97–99.8)	99.4 (98.4–100.0)	**0.01878**	92.5	95	+2.5
Dmean ≥ 99%	99.8 (99.5–100)	99.6 (99.4–99.8)	**0.00036**	90.8	92.5	+1.7
D0.03 cm^3^ ≤ 110%	107.7 (106–108.9)	106.6 (105.8–107.1)	**0.00001**	90.8	97.5	+6.7
V107% ≤ 30%	0.04 (0–0.3)	0 (0–0.01)	**0.00374**	100	100	0
**SVC-PTV**						
V95% ≥ 85%	93 (87.2–96.1)	97.8 (96.3–98.8)	**0.00001**	81.7	100	+18.3
V90% ≥ 90%	97.7 (95.0–99.2)	99.4 (98.6–100)	**0.00001**	91.7	100	+8.3
Dmean ≥ 95%	98.8 (96.9–99.9)	99.1 (98.6–99.7)	**0.0164**	91.7	100	+8.3
D0.03 cm^3^ ≤ 110%	107.0 (105.0–108.2)	107.0 (105.6–108.2)	0.3843	90	95	+5
V107% ≤ 30%	0 (0–0.2)	0 (0–0.15)	0.78716	100	97.5	−2.5
**OARs**						
**Ipsilateral lung**						
D15% ≤ 31 Gy	27.0 (25.7–28.5)	26.30 (25.0–27.6)	**0.00362**	100	99.2	−0.8
D20% ≤ 26.4 Gy	23.8 (22.9–25.0)	23.25 (22.0–24.4)	**0.00672**	96.7	99.2	+2.5
D35% ≤ 17.6 Gy	16.0 (15.0–17.0)	15.3 (14.3–16.5)	**0.00452**	96.7	99.2	+2.5
D50% ≤ 13 Gy	11.5 (10.2–12.0)	11.2 (9.8–12.0)	0.06288	99.2	98.3	−0.9
**Contralateral lung**						
D20% ≤ 13 Gy	6.7 (5.2–8.1)	6.1 (5.3–7.3)	0.08012	100	100	0
D35% ≤ 10.6 Gy	4.7 (4.0–6.0)	4.7 (3.7–5.4)	0.29372	100	100	0
D50% ≤ 9 Gy	3.1 (2.3–4.0)	3.4 (2.4–4.1)	0.90448	100	98.3	−1.7
**Contralateral breast**						
D15% ≤ 17.6 Gy	7.5 (6.0–9.1)	7.6 (6.3–8.6)	0.58232	100	100	0
D20% ≤ 9 Gy	6.5 (5.6–7.7)	6.5 (5.7–7.3)	0.39532	98.3	97.5	−0.8
D35% ≤ 6 Gy	4.9 (4.0–5.2)	4.7 (4.0–5.1)	0.34212	97.5	97.5	0
D50% ≤ 4.4 Gy	3.9 (3.3–4.0)	3.7 (3.4–4.1)	0.6672	95	97.5	+2.5
**Heart** *						
D15% ≤ 17.6 Gy	12.0 (11.0–13.0)			100	-	
D20% ≤ 13 Gy	10.8 (10.0–11.9)			100	-	
**Heart** **						
D15% ≤ 8 Gy	6.1 (5.6–7.0)	6.3 (5.6–7.0)	0.75656	100	97.5	−2.5
D20% ≤ 6 Gy	5.1 (4.5–5.9)	5.1 (4.6–5.7)	0.88076	97.9	93.3	−4.6
Dmean ≤ 5 Gy	4.2 (3.8–4.7)	4.5 (3.9–4.8)	0.12356	94.9	94.2	−0.7
**Brachial plexus**						
D0.03 cm^3^ ≤ 39.6 Gy	38.8 (38.0–39.3)	39.1 (38.9–39.4)	**<0.00001**	94.2	96.7	+2.5
**Spinal cord**						
D0.03 cm^3^ ≤ 17 Gy	13.4 (3.0–15.0)	15.0 (13.7–15.9)	**<0.00001**	98.3	100	+1.7
**Stomach**						
Dmax ≤ 9 Gy	4.4 (2.2–8.8)	5.7 (3.0–9.9)	0.08012		70	
Dmean ≤ 2.6 Gy	1.3 (1.0–2.6)	1.2 (0.7–2.0)	0.00854		91.7	
**Liver**						
V13Gy ≤ 17%	3.7 (0.5–7.5)	3.0 (0.1–6.8)	0.24604	-	100	
Dmean ≤ 4.4 Gy	3.2 (1.6–4.1)	3.0 (1.5–4.0)	0.4965	-	88.3	
**Esophagus** ^§^						
Dmax ≤ 10 Gy (2015) Dmax ≤ 15 Gy (2020)	7.9 (6.9–9.0)	14.8 (14.0–15.7)	**<0.00001**	91.2	60.8	−30.4
**Humeral head** ^‡^						
Dmax ≤ 30 Gy	25.0 (19.0–28.0)	26.7 (24.0–28.6)	0.0012	97.3	94.2	−3.1

* Heart dose relative to the first 22 patients treated between May 2012 and April 2013. ** Heart dose constraints relative to the remaining patients. ^§^ The esophagus dose constraint was evaluated on 113 plans in the 2015 group. ^‡^ Humeral head dose distribution was evaluated on 110 plans in the 2015 group.

**Table 3:** Patients capsular contracture according to Baker classification.

Baker Capsular Contracture	1	2	3	4
2012–2015 plans * n, (%)	3/84 (3.6)	33/84 (39.3)	34/84 (40.5)	12/84 (14.3)
2019–2020 plans n, (%)	7/62 (11.3)	28/62 (45.2)	24/62 (38.7)	3/62 (4.8)

* two patients with no capsular contracture.

**Table 4:** Summary of patients characteristics for the both cohorts.

	2019–2020		2012–2015	
Age (Years)	n	%	n	%
<50	55	45.8	76	63.3
>50	65	54.2	44	36.7
BMI (kg/m^2^)				
Underweight (<18.5)	7	5.8	3	2.5
Normal weight (18.5–24.99)	75	62.5	87	72.5
Overweight (25–29.99)	25	20.8	22	18.3
Obese (>30)	10	8.3	8	6.7
Missing	3	2.5	0	0.0
Irradiated side				
Right side	56	46.7	69	57.5
Left side	64	53.3	51	42.5
Breast reconstruction				
Tissue expander	87	70.8	69	57.5
Prosthesis	33	26.7	51	42.5
Arterial hypertension				
No	104	86.7	106	88.3
Yes	16	13.3	14	11.7
Surgery				
Mastectomy	1	0.8	2	1.7
Mastectomy + SNB	18	15.0	5	4.2
Mastectomy + ALND + SNB	97	80.8	103	85.8
Other	4	3.3	10	8.3
Adjuvant therapy				
HT alone	45	37.5	15	12.5
AC/EC (+HT)	56	46.7	91	75.8
Other CT (+HT)	12	10.0	14	11.7
No	7	5.8	7	5.8
Target therapy				
No	98	81.7	93	77.5
Yes	22	18.3	27	22.5
Bolus				
No	117	97.5	116	96.7
Yes	3	2.5	4	3.3

## Data Availability

The data presented in this study are available on request from the corresponding author.

## List of Abbreviations

ACROP	Advisory Committee for Radiation Oncology Practice
AIRO	Italian Association of Radiotherapy and Clinical Oncology
BC	Breast Cancer
CC	Capsular Contracture
CTV	Clinical Target Volume
CW	Chest Wall
DVH	Dose–Volume Histograms
ESTRO	European Society for Radiotherapy and Oncology
IBR	Immediate Breast Reconstruction
IMRT	Intensity Modulated Radiotherapy
OARs	Organs At Risk
PI	Permanent Implant
PMRT	Postmastectomy Radiation Therapy
PTV	Planning Target Volume
RT	Radiotherapy
SVC	Supraclavicular Nodal Region
TE	Tissue expander
